# Utilizing the National Early Warning Score 2 (NEWS2) to confirm the impact of emergency department management in sepsis patients: a cohort study from taiwan 1998–2020

**DOI:** 10.1186/s12245-024-00614-4

**Published:** 2024-03-15

**Authors:** Ming-Shun Hsieh, Kuan-Chih Chiu, Amrita Chattopadhyay, Tzu-Pin Lu, Shu-Hui Liao, Chia-Ming Chang, Yi-Chen Lee, Wei-En Lo, Vivian Chia-Rong Hsieh, Sung-Yuan Hu, Chorng-Kuang How

**Affiliations:** 1 Department of Emergency Medicine, Taoyuan Branch, Taipei Veterans General Hospital, Taoyuan, Taiwan; 2 Department of Emergency Medicine, Taipei Veterans General Hospital, Taipei, Taiwan; 3 School of Medicine, National Yang Ming Chiao Tung University, Taipei, Taiwan; 4 Department of Emergency Medicine, Taichung Veterans General Hospital, Taichung, Taiwan; 5 College of Public Health, National Taiwan University, Taipei, Taiwan; 6 Department of Pathology and Laboratory, Taoyuan Branch, Taipei Veterans General Hospital, Taoyuan, Taiwan; 7 Department of Health Services Administration, China Medical University, Taichung, Taiwan; 8 School of Medicine, Chung Shan Medical University, Taichung, Taiwan; 9 Department of Post-Baccalaureate Medicine, College of Medicine, National Chung Hsing University, Taichung, Taiwan

**Keywords:** Hospital mortality, National early warning score 2 (NEWS2), Prediction score, Sepsis, Sequential organ failure assessment score (SOFA score)

## Abstract

**Background:**

Most sepsis patients could potentially experience advantageous outcomes from targeted medical intervention, such as fluid resuscitation, antibiotic administration, respiratory support, and nursing care, promptly upon arrival at the emergency department (ED). Several scoring systems have been devised to predict hospital outcomes in sepsis patients, including the Sequential Organ Failure Assessment (SOFA) score. In contrast to prior research, our study introduces the novel approach of utilizing the National Early Warning Score 2 (NEWS2) as a means of assessing treatment efficacy and disease progression during an ED stay for sepsis.

**Objectives:**

To evaluate the sepsis prognosis and effectiveness of treatment administered during ED admission in reducing overall hospital mortality rates resulting from sepsis, as measured by the NEWS2.

**Methods:**

The present investigation was conducted at a medical center from 1997 to 2020. The NEWS2 was calculated for patients with sepsis who were admitted to the ED in a consecutive manner. The computation was based on the initial and final parameters that were obtained during their stay in the ED. The alteration in the NEWS2 from the initial to the final measurements was utilized to evaluate the benefit of ED management to the hospital outcome of sepsis. Univariate and multivariate Cox regression analyses were performed, encompassing all clinically significant variables, to evaluate the adjusted hazard ratio (HR) for total hospital mortality in sepsis patients with reduced severity, measured by NEWS2 score difference, with a 95% confidence interval (adjusted HR with 95% CI). The study employed Kaplan-Meier analysis with a Log-rank test to assess variations in overall hospital mortality rates between two groups: the “improvement (reduced NEWS2)” and “non-improvement (no change or increased NEWS2)” groups.

**Results:**

The present investigation recruited a cohort of 11,011 individuals who experienced the first occurrence of sepsis as the primary diagnosis while hospitalized. The mean age of the improvement and non-improvement groups were 69.57 (± 16.19) and 68.82 (± 16.63) years, respectively. The mean SOFA score of the improvement and non-improvement groups were of no remarkable difference, 9.7 (± 3.39) and 9.8 (± 3.38) years, respectively. The total hospital mortality for sepsis was 42.92% (4,727/11,011). Following treatment by the prevailing guidelines at that time, a total of 5,598 out of 11,011 patients (50.88%) demonstrated improvement in the NEWS2, while the remaining 5,403 patients (49.12%) did not. The improvement group had a total hospital mortality rate of 38.51%, while the non-improvement group had a higher rate of 47.58%. The non-improvement group exhibited a lower prevalence of comorbidities such as congestive heart failure, cerebral vascular disease, and renal disease. The non-improvement group exhibited a lower Charlson comorbidity index score [4.73 (± 3.34)] compared to the improvement group [4.82 (± 3.38)] The group that underwent improvement exhibited a comparatively lower incidence of septic shock development in contrast to the non-improvement group (51.13% versus 54.34%, *P* < 0.001). The improvement group saw a total of 2,150 patients, which represents 38.41% of the overall sample size of 5,598, transition from the higher-risk to the medium-risk category. A total of 2,741 individuals, representing 48.96% of the sample size of 5,598 patients, exhibited a reduction in severity score only without risk category alteration. Out of the 5,403 patients (the non-improvement group) included in the study, 78.57% (4,245) demonstrated no alteration in the NEWS2. Conversely, 21.43% (1,158) of patients exhibited an escalation in severity score. The Cox regression analysis demonstrated that the implementation of interventions aimed at reducing the NEWS2 during a patient’s stay in the ED had a significant positive impact on the outcome, as evidenced by the adjusted HRs of 0.889 (95% CI = 0.808, 0.978) and 0.891 (95% CI = 0.810, 0.981), respectively. The results obtained from the Kaplan-Meier analysis indicated that the survival rate of the improvement group was significantly higher than that of the non-improvement group (*P* < 0.001) in the hospitalization period.

**Conclusion:**

The present study demonstrated that 50.88% of sepsis patients obtained improvement in ED, ascertained by means of the NEWS2 scoring system. The practical dynamics of NEWS2 could be utilized to depict such intricacies clearly. The findings also literally supported the importance of ED management in the comprehensive course of sepsis treatment in reducing the total hospital mortality rate.

**Supplementary Information:**

The online version contains supplementary material available at 10.1186/s12245-024-00614-4.

## Introduction

Sepsis was a complex syndrome that was induced by severe infection with a subsequent unregulated immune response and multiple organ failures [1]. It was a leading cause of hospital admission and death. Every year, sepsis is estimated to affect approximately 50 million patients and resulted in 11 million deaths globally [2]. 

The latest study conducted in Hong Kong by electronic health records (EHR) reported that in 2018, the age- and sex-adjusted standardized sepsis incidence was 759 per 100,000 between 2009 and 2018 and standardized sepsis mortality was 156 per 100,000 [3]. 

In sepsis, early interventions were the key to survival, which warranted structured approaches to prevent early mortality. Medical management in the ED is a critical step in the sepsis treatment course, emphasizing fluid resuscitation, infection source control, respiratory support, and dedicated nursing care [4–6]. While the significance of this phase (ED diagnoses and treatments) was widely recognized, few studies have quantitatively evaluated the impact of ED interventions on sepsis outcomes, particularly in expansive cohorts. Our study spanned crucial transitions in sepsis guidelines, incorporating phases from SIRS to Sepsis III. The breadth of our analysis offers insights unmatched by prior research. Conducted in Taiwan from 1997 to 2020, this cohort study leveraged an extensive hospital-based database to elucidate the role of the ED in managing sepsis. Patients were assessed using the National Early Warning Score 2 (NEWS2) during their ED stay. We chose NEWS2 because it eliminated the need for repeated blood tests and delivered swift and objective evaluations. Furthermore, its efficacy has been confirmed in numerous studies [7–9]. 

The National Early Warning Score (NEWS), introduced by the Royal College of Physicians in London, stands out as one of the most renowned Early Warning Scores (EWS) due to its strong prognostic capabilities. In 2017, in light of more expansive validation datasets covering diverse patient categories, the Royal College refined it to NEWS2, enhancing its precision. The NEWS2 score is pivotal in pinpointing patients susceptible to clinical decline, necessitating prompt medical intervention. Widely adopted in healthcare settings, this measure aims to bolster patient safety and elevate treatment outcomes. A NEWS2 score of 5 or above is a critical marker, signaling an urgent need for response in sepsis cases [10]. 

In EDs, NEWS2 outperforms the quick Sequential Organ Failure Assessment (qSOFA) in identifying sepsis accompanied by organ dysfunction, the need for intensive care, and mortality due to infection [11]. Utilizing these scores has fostered an alert system that promptly identifies and prioritizes sepsis patients at elevated risk of critical illness, ensuring they receive care in line with the sepsis bundles [12]. 

Although multiple scoring systems had been utilized to predict the hospital outcome of sepsis patients with the varied sensitivity and specificity. However, there was no large cohort study currently which utilized the NEWS2, to simply and precisely present the impact of ED management to sepsis patients. So, in the current study, we aimed to present the utilization of NEWS2 scoring system in demonstrating the severity difference of the sepsis patients between the ED arrival and discharge, and to predict the hospital mortality. That is, the endpoint of this study was the total hospital mortality rate which was related to the ED management and was predicted by the NEWS2 scoring system with associated statistical models.

## Methods

### Study population

**Fig. 1:**
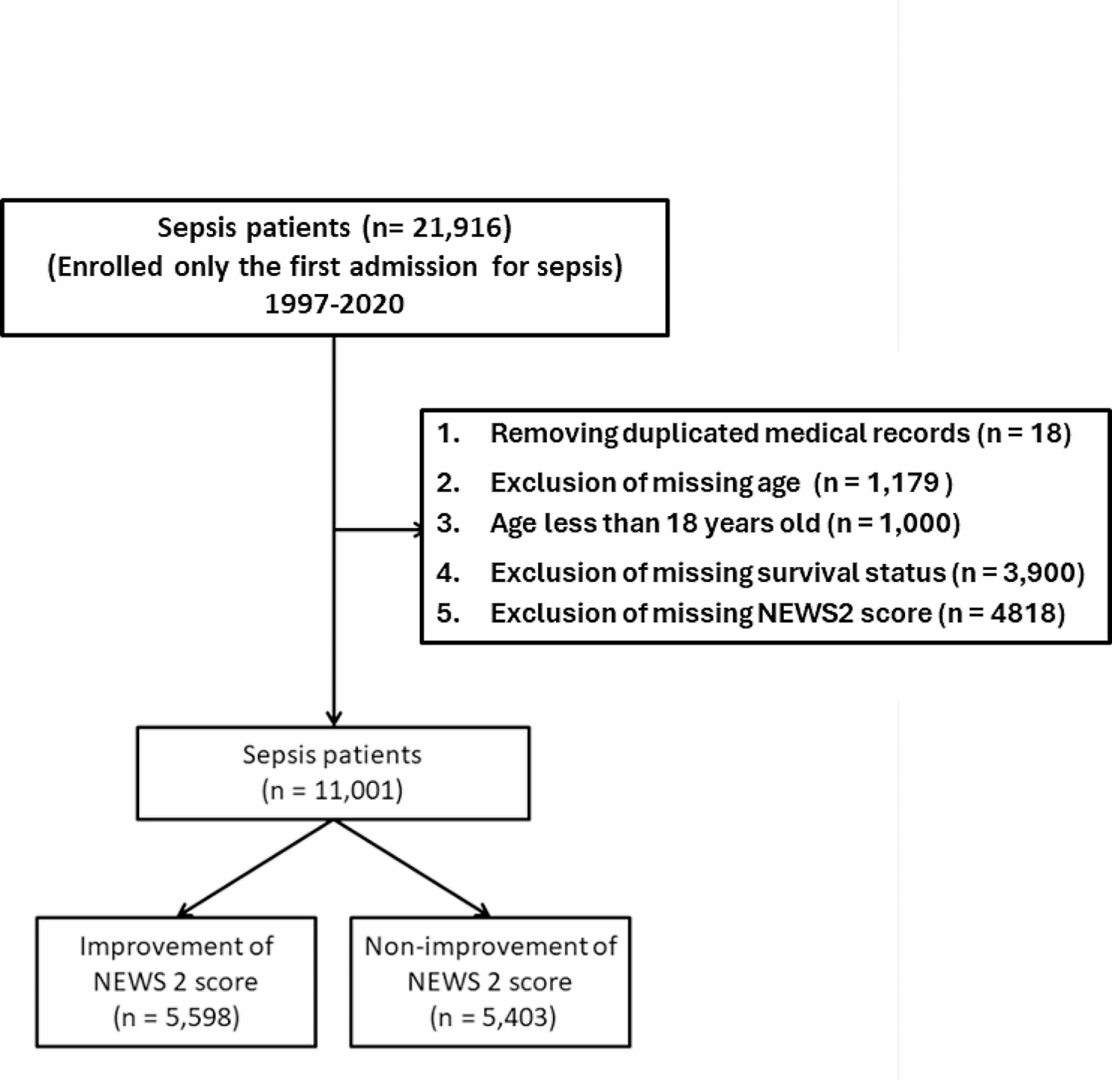
Selection algorithm

This study analyzed data prospectively collected from 1997 to 2020, comprising a continuous cohort of sepsis patients presenting to the ED of a central Taiwanese medical center [13]. For each patient, the NEWS2 score was derived from the initial and final readings recorded during their ED visit for sepsis treatment. Exclusions encompassed patients transferred from other hospitals, including those via their EDs. Notably, only the first episode of sepsis was included for each participant based on historical data. Our dataset comprised 21,926 patients, each diagnosed with sepsis as the primary condition on both their ED and hospital admission records. The patients with missing survival status (*n* = 3,900) or NEWS2 score (4,818) were also excluded. The patient selection process is delineated in Fig. 1.

### Study variables

Demographic information, comorbidities, Charlson comorbidity index score (CCI score), septic shock, hospital mortality, SOFA score, NEWS2 (initial, final, and the difference in ED), length of ED stay, ICU admission, procedures, main infection site, and laboratory data, including procalcitonin and lactate level, were collected from all study participants [14, 15]. Continuous variables were reported as mean ± standard deviation (SD), while categorical variables were described as the number and percentage of total subjects.

The NEWS2 scoring system aggravated each scoring of the following seven physiologic parameters including (1) respiratory rate, (2) SpO2, (3) air or oxygen use, (4) systolic blood pressure, (5) pulse rate, (6) consciousness, and (7) temperature to aggravate into a sum of the total NEWS2 scores. According to the NEWS2 scores, the clinical risk was classified into three categories, that is, (1) the Low-risk category (aggravated NEWS2 score = 0–4) (2) the Medium-risk category (aggravated NEWS2 score = 5–6 or individual parameter scoring = 3), and (3) the High-risk category (aggravated NEWS2 score = or > 7). The sepsis patients were therefore divided into two groups: (1) the improvement group (defined as “improvement in risk category” and “no risk category change but decreased score”) and (2) the non-improvement group (defined as “no change of NEWS2” and “deterioration”).

### Ethics approval and informed consent

The study was carried out in accordance with the STROBE reporting guidelines for observational studies [16]. This study was approved by the institutional review board of Taichung Veterans General Hospital (IRB No. CE22240B). Due to the anonymization of patient identification to safeguard privacy prior to data dissemination, the study was deemed exempt from obtaining informed consent from participants.

### Statistical analysis

The study utilized statistical tests such as the student t-test for continuous variables and chi-square test for categorical variables to compare clinical characteristics between the improvement and non-improvement groups. Univariate and multivariate Cox regression analyses were conducted on all variables to determine the adjusted hazard ratio with a 95% confidence interval (adjusted HR with 95% CI) for total hospital mortality in patients with sepsis. Our study centered on the influence of ED management on overall hospital mortality as determined by the NEWS2.

In the Cox regression model with clinical adjustment, we conducted three type of multi-variate regressions, that is, putting (1) the variables of P value in significance in the crude model, (2) all clinically significant variables, and (3) the variables excluding laboratory data. We mainly presented the result in model 3 where age, sex, for age, sex, comorbidities, septic shock, improvement of NEWS2 severity, length of ED stay and ICU admission. The “improvement of NEWS2 severity” were divided into (1) no change in risk category but decreased NEWS2 severity (served as the reference in the Cox regression model), (2) high to medium, (3) high to low, and (4) medium to low categories, respectively [10]. 

Stratification analyses were conducted according to (1) the age, that is, 18–45, 46–65, 66–85, and more than 85 years and (2) the primary infection sites were also classified into central nervous, respiratory, cardiovascular, gastrointestinal/biliary tract, genitourinary, soft tissue/ musculoskeletal systems, and device related.

A Kaplan–Meier analysis with a two-tailed Log-rank test was performed to determine the efficacy of ED management on total hospital mortality (28-day) between the “improvement (reduced NEWS2)” and “non-improvement (no score change or increased NEWS2)” groups. A P value < 0.05 was used to define statistical significance. All analyses were conducted using R, whereas the Kaplan-Meier analysis was conducted using the Survival package.

## Results

The present investigation recruited a cohort of 11,011 individuals who experienced the first occurrence of sepsis as the primary diagnosis while hospitalized. The present investigation recruited a cohort of 11,011 individuals who experienced the first occurrence of sepsis as the primary diagnosis while hospitalized. The mean age of the improvement and non-improvement groups were 69.57 (± 16.19) and 68.82 (± 16.63) years (*P* = 0.02), respectively. The mean SOFA score of the improvement and non-improvement groups were of no remarkable difference, 9.7 (± 3.39) and 9.8 (± 3.38), respectively.

Following treatment in accordance with the prevailing guidelines at that time, a total of 5,598 out of 11,011 patients (50.84%) demonstrated improvement in the NEWS2, while the remaining 5,403 patients (49.12%) did not. In other words, there are 50.84% (5,598) of patients got benefit from the care of emergency medicine. (Table 1)

**Table 1:** The difference in NEWS2 between the improvement and non-improvement groups

Variables	Improvement in the NEWS2 (*n* = 5,598, 50.84%)	Non-improvement in the NEWS2 (*n* = 5,403, 49.16%)	P^1^
**Demographic characteristics**			
Age [mean (SD)]	69.57 (16.19)	68.82 (16.63)	0.02*
Sex [Male, n (%)]	3506 (62.63)	3398 (62.89)	0.79
**Mortality [n (%)]**	2156 (38.51)	2571 (47.58)	< 0.001***
**Comorbidity [n (%)]**			
AMI	434 (7.75)	450 (8.33)	0.28
CHF	1022 (18.26)	900 (16.66)	0.03*
Peripheral vascular disease	389 (6.95)	341 (6.31)	0.19
Cerebrovascular disease	1593 (28.46)	1391 (25.74)	< 0.01**
Dementia	633 (11.31)	571 (10.57)	0.23
COPD	1639 (29.28)	1518 (28.10)	0.18
Rheumatic disease	327 (5.84)	317 (5.87)	0.99
Peptic ulcer disease	1755 (31.35)	1637 (30.30)	0.24
Mild liver disease	623 (11.13)	586 (10.85)	0.66
Diabetes without chronic complication	1296 (23.15)	1234 (22.84)	0.71
Diabetes with chronic complication	710 (12.68)	621 (11.49)	0.06
Hemiplegia or paraplegia	178 (3.18)	186 (3.44)	0.47
Renal disease	2023 (36.14)	1837 (34)	0.02*
Malignancy, including lymphoma and leukemia	1452 (25.94)	1370 (25.36)	0.50
Moderate or severe liver disease	409 (7.31)	438 (8.11)	0.12
Metastatic solid tumor	1205 (21.53)	1198 (22.17)	0.42
Charlson comorbidity index (CCI, mean (SD))	4.82 (3.38)	4.73 (3.34)	0.15
SOFA score (mean (SD))	9.7 (3.39)	9.80 (3.38)	0.10
Septic shock (n (%))	2862 (51.13)	2936 (54.34)	< 0.001***
**NEWS 2 (mean (SD))**			
First	8.76 (2.42)	6.47 (1.91)	< 0.001***
Last	6.08 (1.87)	7.50 (2.26)	< 0.001***
**NEWS2 difference**			
**(1) Improvement**	5,598 (100)		
Improvement in risk category	2857 (51.04)		
(i) High to medium	2150 (38.41)		
(ii) High to low	367 (6.56)		
(iii) Medium to low	340 (6.07)		
No risk category change but decreased score	2741 (48.96)		
**(2) Non-improvement**		5403 (100)	
No change of NEWS2		4245 (78.57)	
Deterioration		1158 (21.43)	
Length of ED stay (hours, mean (SD))	35.1 (42.08)	32.85 (43.11)	< 0.01**
ICU admission	2550 (45.55)	2531 (46.84)	0.18
**Primary Infection site (n (%))**			
Central nervous	53 (0.95)	58 (1.07)	0.57
Respiratory	2309 (41.25)	2149 (39.77)	0.12
Cardiovascular	65 (1.16)	55 (1.02)	0.53
Gastrointestinal/Biliary tract	1046 (18.69)	1049 (19.42)	0.34
Genitourinary	1687 (30.14)	1555 (28.78)	0.12
Soft tissue/musculoskeletal	207 (3.70)	196 (3.63)	0.88
**Laboratory data (mean (SD))**			
WBC (/µL)	13,810 (12,507)	13,414 (13,426)	0.11
Hemoglobin (g/dL)	11.36 (2.64)	11.47 (2.58)	0.03*
Platelet (×10^3^/µL)	202 (127)	200 (122)	0.25
Albumin (g/dL)	2.77 (0.64)	2.78 (0.65)	0.36
Total Bilirubin (mg/dL)	1.54 (3.06)	1.73 (3.64)	< 0.01**
Creatinine (mg/dL)	2.14 (2.04)	2.12 (2.06)	0.53
CRP (mg/dL)	12.11 (10.19)	11.60 (10)	0.01*
Procalcitonin (ng/mL)	15.33 (27.59)	15.30 (28.72)	0.97
Lactate (mmol/L)	3.51 (3.14)	3.13 (3.13)	< 0.001***

^1^Student t-test for continuous variables and chi-square test for categorical variables.

*P < 0.05, **P < 0.01, ***P < 0.001

**Abbreviations**:

AMI: Acute myocardial infarction; CCI: Charlson comorbidity index; CHF: Congestive heart failure; COPD: Chronic obstructive pulmonary disease; CRP: C-reactive protein; ED: Emergency department; ICU: Intensive Care Unit; NEWS2: National Early Warning Score 2; SD: standard deviation; SOFA score: Sequential organ failure assessment score.

The total hospital mortality for sepsis was 42.92% (4,727/11,011). The improvement group had a total hospital mortality rate of 38.51%, while the non-improvement group had a higher rate of 47.58%.

The non-improvement group exhibited a lower prevalence of comorbidities such as congestive heart failure, cerebral vascular disease, and renal disease. The non-improvement group exhibited a lower Charlson comorbidity index score [4.73 (± 3.34)] compared to the improvement group [4.82 (± 3.38)]. The group that underwent improvement exhibited a comparatively lower incidence of septic shock development compared to the non-improvement group (51.13% versus 54.34%, *P* < 0.001).

Patients were classified into “higher” or “lower” CCI score categories based on the mean CCI score. In the group with NEWS2 improvement, the hospital mortality rate for patients with a higher CCI score was 46.38%, whereas those with a lower CCI score had a mortality rate of 30.45% (*P* < 0.05). Similarly, in the non-improvement group, patients with a higher CCI score exhibited a mortality rate of 56.50%, in contrast to the 38.72% rate observed in those with a lower CCI score (*P* < 0.05) (Supplementary Table 1).

In the group that showed NEWS2 improvement, septic shock patients had a hospital mortality rate of 55.52%, significantly higher than the 20.72% rate observed in non-septic shock patients (*P* < 0.05). Similarly, in the non-improvement group, the mortality rate was 64.58% for septic shock patients, in contrast to 38.72% for those without septic shock (*P* < 0.05) (Supplementary Table  2).

The improvement group saw a total of 2,150 patients, which represents 38.41% of the overall sample size of 5,598, transition from the higher-risk to the medium-risk category. A total of 2,741 individuals, representing 48.96% of the sample size of 5,598 patients, exhibited a reduction in severity score only without risk category alteration. Out of the 5,403 patients (the non-improvement group) included in the study, 78.57% (4,245) demonstrated no alteration in the NEWS2. Conversely, 21.43% (1,158) of patients exhibited an escalation in severity score.

The group that demonstrated improvement experienced a comparatively longer duration of stay in the ED, with a mean of 35.10 (± 42.08) hours, in contrast to the non-improvement group, whose mean duration of stay was 32.85 (± 43.11) hours (*P* = 0.006).

There are 50.84% (5,598) patients got benefit from the care of emergency medicine in general. Improvement in risk category, including high to medium, high to low, and medium to low were 38.41%, 6.56%, and 6.07% respectively. And there were 2741 patients got decreased risk score only without category change.

The Cox regression analysis demonstrated that the implementation of interventions aimed at reducing the NEWS2 during a patient’s stay in the ED had a significant positive impact on the outcome, as evidenced by the adjusted HRs of 0.889 (95% CI = 0.808, 0.978) (model 2). (Table 2)

**Table 2:** Impact of “improvement of NEWS2 score” in sepsis patients in ED on the hospital mortality by Cox regression models

Variables	Mortality							
	Crude HR (95% CI)	Crude P		Model 1 ^#^Adjusted HR (95% CI)	*P* value		Model 2 ^#^Adjusted HR (95% CI)	*P* value
**Improvement of NEWS2 score**	0.84 (0.80, 0.89)	< 0.001***		0.89 (0.81, 0.98)	0.02*		0.89 (0.81, 0.98)	0.02*
**Demographic characteristics**								
Age	1.00 (1.00, 1.00)	< 0.001***		1.01 (1.01, 1.01)	< 0.001***		1.01 (1.01 1.02)	< 0.001***
Sex	1.13 (1.06, 1.19)	< 0.01**		1.04 (0.94, 1.15)	0.49		1.05 (0.94, 1.16)	0.38
**Comorbidity**								
AMI	1.12 (1.01, 1.23)	0.03*		1.13 (0.96, 1.34)	0.13		1.13 (0.95, 1.33)	0.16
CHF	1.04 (0.97, 1.12)	0.22					1.10 (0.97, 1.25)	0.14
Peripheral vascular disease	0.95 (0.85, 1.06)	0.41					0.93 (0.77, 1.12	0.42
Cerebrovascular disease	0.82 (0.77, 0.88)	< 0.001***		0.88 (0.78, 0.98)	0.03*		0.88 (0.78, 0.99)	0.04
Dementia	0.88 (0.80, 0.97)	0.01*		0.87 (0.73, 1.04)	0.13		0.88 (0.74, 1.05	0.15
COPD	0.93 (0.87, 0.99)	0.01*		0.92 (0.82, 1.03)	0.15		0.91 (0.82, 1.04)	0.12
Rheumatic disease	0.93 (0.83, 1.05)	0.26					1.14 (0.96, 1.35)	0.12
Peptic ulcer disease	0.99 (0.94, 1.06)	0.96					0.93 (0.83, 1.03)	0.18
Mild liver disease	1.11 (1.01, 1.20)	0.01*		1.03 (0.88, 1.19)	0.71		1.05 (0.90, 1.22)	0.53
Diabetes without chronic complications	0.94 (0.87, 1.00)	0.08					0.89 (0.79, 1.01)	0.06
Diabetes with chronic complications	0.87 (0.80, 0.96)	0.01*		0.91 (0.78, 1.06)	0.22		0.88 (0.75, 1.03)	0.11
Hemiplegia or paraplegia	0.81 (0.68, 0.96)	0.02*		0.98 (0.73, 1.30)	0.87		0.96 (0.72 1.27)	0.76
Renal disease	1.08 (1.02, 1.14)	0.01*		1.02 (0.91, 1.14)	0.76		1.01 (0.89, 1.13)	0.88
Malignancy, including lymphoma and leukemia	1.09 (1.03, 1.16)	0.01*		1.22 (1.08, 1.36)	0.01*		1.24 (1.11 1.39)	< 0.01**
Moderate or severe liver disease	1.82 (1.67, 1.99)	< 0.001***		1.49 (1.24, 1.79)	< 0.001***		1.55 (1.29, 1.86)	< 0.001***
Metastatic solid tumor	1.70 (1.59, 1.81)	< 0.001***		1.61 (1.42, 1.83)	< 0.001***		1.64 (1.45, 1.85)	< 0.001***
Septic shock	1.68 (1.57, 1.80)	< 0.001***		1.64 (1.41, 1.91)	< 0.001***		1.60 (1.41, 1.91)	< 0.001***
ICU admission	0.83 (0.78, 0.88)	< 0.001***		0.69 (0.60, 0.78)	< 0.001***		068 (0.62, 0.78)	< 0.001***
Length of ED stay (hours)	0.99 (0.99, 1.00)	< 0.001***		0.99 (0.99, 1.00)	0.04*		0.99 (0.99 0.99)	0.04*
**Laboratory data**								
WBC (/µL)	1 (1.00, 1.00)	0.04*		1 (1.00, 1.00)	< 0.01**		1 (1.00, 1.00)	< 0.01**
Hemoglobin (g/dL)	0.95 (0.94, 0.96)	< 0.001***					0.98 (0.96, 1.01)	0.19
Platelet (×10^3^/µL)	0.99 (0.99, 0.99)	< 0.001***		0.99 (0.99, 0.99)	< 0.001***		0.99 (0.99, 1.00)	< 0.001***
Albumin (g/dl)	0.75 (0.72, 0.79)	< 0.001***		0.78 (0.72, 0.85)	< 0.001***		0.77 (0.71, 0.84)	< 0.001***
Total Bilirubin (mg/dL)	1.05 (1.04, 1.05)	< 0.001***		1.03 (1.02, 1.05)	< 0.001***		1.03 (1.02, 1.05)	< 0.001***
Creatinine (mg/dL)	1.04 (1.03, 1.05)	< 0.001***					1.02 (0.99, 1.05)	0.08
CRP (mg/dL)	1.00 (0.99, 1.01)	0.12					1.00 (0.99, 1.01)	0.53
Procalcitonin (ng/mL)	1.00 (1.00, 1.00)	< 0.01**					1.00 (0.99, 1.00)	0.12
Lactate (mmol/L)	1.01 (1.01, 1.01)	< 0.001***		1.01 (1.00, 1.01)	< 0.001***		1.01 (1.01, 1.01)	< 0.001***

*P < 0.05, **P < 0.01, ***P < 0.001

**Abbreviations**:

AMI: Acute myocardial infarction; CHF: Congestive heart failure; CI: confidence interval; COPD: Chronic obstructive pulmonary disease; HR: Hazard ratio; ED: Emergency department: ICU: Intensive care unit; CRP: C-reactive protein

Model 1^#^: adjusted for the variables of significance (*P* < 0.05) in the univariate analysis

Model 2^#^: adjusted for all the variables of clinical importance (all in univariate analysis).

**Fig. 2:**
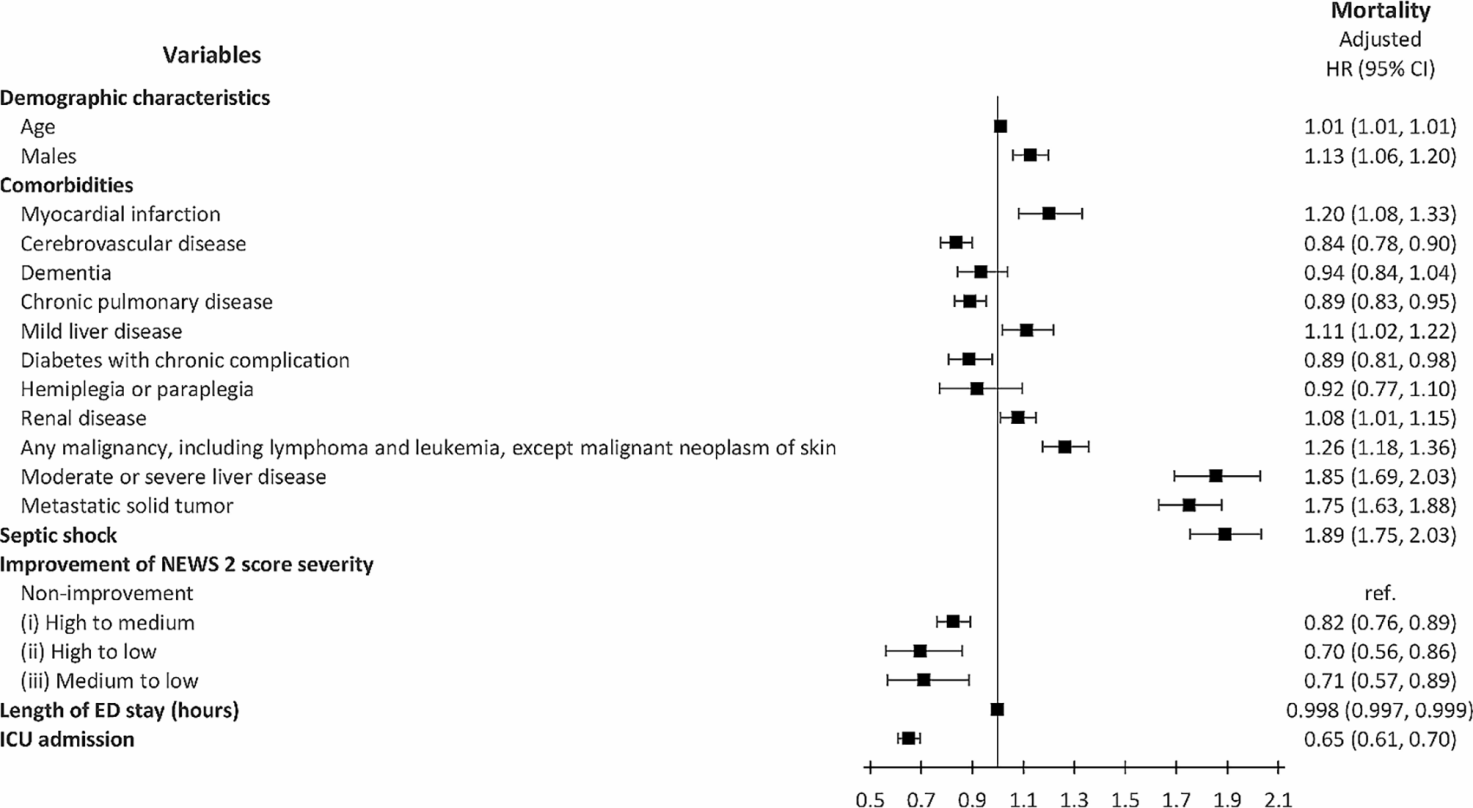
Impact of NEWS2 score difference in the ED to the hospital mortality by Cox regression model

In the further analyses by the Cox regression model, the study demonstrated that (1) high to medium, (2) high to low, and (3) medium to low categories had decreased mortality rate of (1) adjusted HR = 0.82 (95% CI: 0.76–0.89), (2) adjusted HR = 0.70 (95% CI: 0.56–0.86), and (3) adjusted HR = 0.71 (95% CI: 0.57–0.89), respectively, when putting the “no change in risk category but decreased NEWS2 severity” into the Cox regression model as the reference. (Fig. 2) The above description made it clear that ED management literally help the sepsis patients during the ED stay. And the length of ED did not increase the risk of mortality rate (adjusted HR = 0.998 (95% CI: 0.997–0.999)). The manifestation of septic shock exhibited a significant correlation with increased hospital mortality, as indicated by the adjusted HR of 1.89 (95% CI: 1.75–2.03).

In the subgroup analyses, the adjusted HR with 95% CI for the four age groups (18–45, 46–65, 66–85 and > 85 years) were 0.802 (95% CI: 0.628–1.026), 0.823 (95% CI: 0.739–0.917), 0.873 (95% CI: 0.802–0.949), and 0.923 (95% CI: 0.807–1.054), respectively. (Supplement Fig. 01) The age between 46 and 65 and 66–85 group significantly benefit more from improved severity based on NEWS2 during ED stay.

In the subgroup analysis according to the primary infection site, the adjusted HR with 95% CI were 0.864 (95% CI: 0.462–1.617) for central nervous system, 0.888 (95% CI: 0.818–0.963) for respiratory system, 0.818 (95% CI: 0.466–1.437) for cardiovascular system, 0.871 (95% CI: 0.768–0.988) for gastrointestinal/biliary tract, 0.840 (95% CI: 0.727–0.972) for genitourinary system, 0.788 (95% CI: 0.548–1.134) for soft tissue/musculoskeletal system, and 1.019 (95% CI: 0.646–1.608) for device-related infection, respectively. (Supplement Fig. 02)

**Fig. 3:**
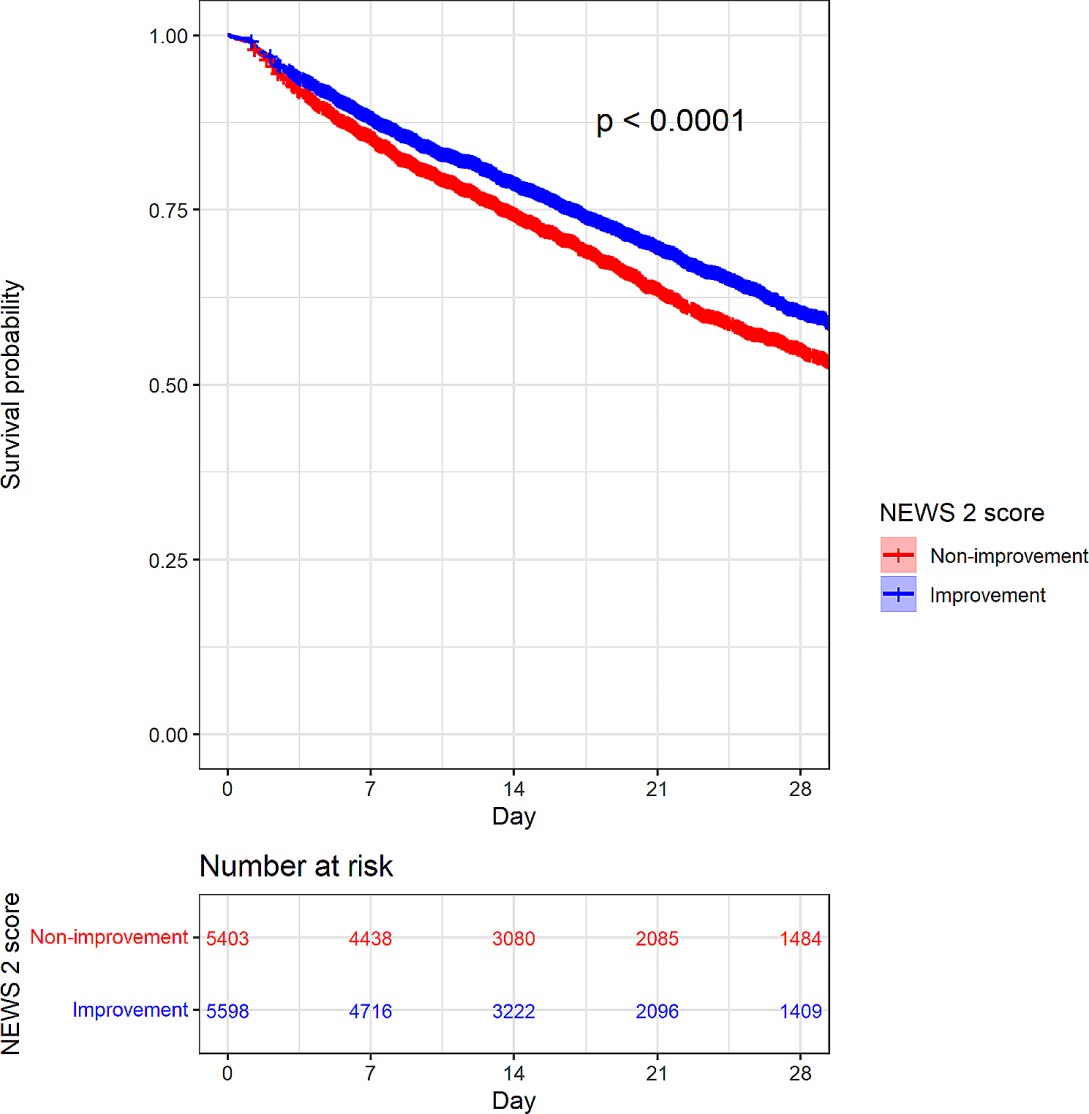
A Kaplan–Meier analysis with Log-rank test to determine the efficacy of ED management on total hospital mortality between the “improvement (reduced NEWS2)” and “non-improvement groups (no score change or increased NEWS2)

The results obtained from the Kaplan-Meier analysis indicated that the survival rate of the improvement group was significantly higher than that of the non-improvement group (*P* < 0.001) in the hospitalization period. (Fig. 3)

## Discussion

The present investigation represents the first attempt to employ the NEWS2 metric to demonstrate the beneficial influence of ED interventions on sepsis outcomes, specifically with a substantial sepsis patient population spanning the years 1997 to 2020. The study revealed that 50% of the sepsis patients responded favorably to treatment in the ED, while the remaining 50% did not. The improvement of NEWS2 score in this context acted as a proxy for ameliorating the clinical state throughout the ED sojourn, irrespective of the duration of the stay. At least half of the enrollees benefit from ED care for sepsis. Rather than being the first initial aid in sepsis treatment, the ED plays an important role than that in our stereotypic understanding.

Here, we have extended the application of the NEWS2 scoring system in clinical settings. The most recent iteration of the National Early Warning Score (NEWS) is NEWS2. Initially developed in 2012 and subsequently revised in December 2017, NEWS2 proposes a framework for standardizing the evaluation and management of acute illness. In this study, the implementation of NEWS2 has standardized the evaluation of the severity of acute illness.

The NEWS2 operates on an aggregate scoring mechanism that allocates scores to physiological measurements, which are conventionally documented upon patient admission or during monitoring in a hospital environment. This scoring mechanism is underpinned by six fundamental physiological parameters: respiration rate, oxygen saturation, systolic blood pressure, pulse rate, level of consciousness or emergent confusion, and temperature. Notably, the NEWS2 scoring framework demonstrates enhanced sensitivity in identifying nuanced but vital alterations in patients within a clinical context.

In most studies executed within the ED, vital signs have typically been measured on a singular occasion, predominantly during the triage phase [17]. It is imperative to note that the clinical status of a patient might undergo significant alterations during their tenure in the ED, changes which are often manifest in the fluctuations of vital signs. While Early Warning Systems (EWS), such as the NEWS2, frequently rely on a solitary assessment of clinical parameters, their precision in prognosticating hospital outcomes, predominantly defined as hospital mortality, can be constrained. However, the acuity of the NEWS2 system in discerning clinical deterioration, particularly with scores exceeding 5, endows it with undeniable clinical pertinence and utility.

In the research conducted by Vincent et al., it was elucidated that approximately 20% of patients admitted to the ED with presumptive infection or sepsis exhibited deterioration within the initial 72 h post-admission. Contrarily, our dataset indicated that over half of the patients diagnosed with sepsis showed signs of improvement when admitted through the ED. This observed improvement can be attributed to several factors: the timely detection of sepsis, appropriate resuscitative measures such as fluid replenishment and the employment of vasopressors/inotropes, expedited acquisition of laboratory data, prompt administration of antibiotics, and multifaceted support including respiratory assistance. Such interventions, collectively referred to as sepsis bundle care, have proven instrumental in stabilizing patients within a constrained temporal framework.

In a 2022 study by Nielsen et al., it was observed that numerous patients, initially presenting without score positivity (i.e., NEWS2 score < 5), experienced deterioration, subsequently achieving score positivity (NEWS2 ≥ 5) within the initial 4 h of their ED stay [18]. In contrast, our investigation revealed that the preliminary NEWS2 scores for the improvement and non-improvement cohorts were, on average, greater than 8 and greater than 6, respectively, upon initial evaluation in the ED. Notably, after durations exceeding 35 h and 32 h for the respective groups within the ED, a mere 10.52% (1,158/11,011) exhibited deterioration, while 38.55% (4,245/11,011) remained clinically stable without any evident alterations in their condition.

Examining the disparity between the initial and final vital sign measurements in the ED provides valuable insights into clinical progression, as the objective was primarily to assess the efficacy of ED management and to discern potential clinical deterioration. It should be noted, however, that such an evaluation was not aimed at providing an exact prediction of eventual hospital mortality, given the prolonged duration which might not solely be influenced by ED interventions. For more rigorous monitoring, we advocate for periodic vital sign assessments and sepsis scoring, as these can enhance the precision of scores and facilitate the early detection of deterioration.

In comparison to alternative scoring systems, including the Modified Early Warning Score (MEWS), the SOFA score, and the Mortality in Emergency Department Sepsis score (MEDS score), NEWS2 offers the advantage of not necessitating repeated blood tests, such as those for lactate levels. Notably, NEWS2 abstains from incorporating ambiguous parameters. For instance, SpO2 presents challenges in discerning whether measurements were taken with supplemental oxygen or in ambient air. Furthermore, the Glasgow Coma Scale (GCS) can yield varied evaluations when assessed by different individuals simultaneously; it’s also worth noting that GCS was originally formulated to gauge consciousness levels post head injury, rather than for the evaluation of sepsis.

Within our research parameters, a notable decrease in the hospital mortality rate was documented, descending from 48.88% in 2009 to 37.01% in 2015. Contrarily, there was an unexpected ascent to 43.49% in 2016, which subsequently receded to 42.84% by 2020. This inflection in 2016 could potentially be attributed to the paradigm shift in sepsis definitions, transitioning from SIRS to Sepsis-3. In contrast, the meta-analysis by Luhr et al., which encompassed 44 randomized clinical trials from 2002 to 2016, presented varied results [19]. The data extrapolated from these randomized controlled trials (RCTs) showcased a discernible decline in the 28-day mortality rate for patients diagnosed with severe sepsis and septic shock. Yet, post-severity adjustment at the study’s inception, the temporal progression revealed no statistically meaningful fluctuation in mortality rates. It remains imperative to approach any comparisons of hospital mortality rates with circumspection, given the plausible divergences in study cohorts or demographics across distinct medical institutions.”

In a study conducted by the Emergency Medicine Shock Research Network Investigators, patients were categorized into two cohorts (1) Lactate Clearance Group: Those in which lactate decreased by 10% or more from its initial value, or where both initial and subsequent levels were ≤ 2.0 mmol/L. (2) Lactate Non-Clearance Group: Those in which the repeat lactate levels reduced by less than 10% from the initial measurement [20].

Hospital mortality rates were significantly different between the two groups, with the Lactate Non-Clearance Group exhibiting a 60% rate compared to the 19% observed in the Lactate Clearance Group. Lactate non-clearance emerged as an independent prognostic factor for mortality, with an adjusted odds ratio (OR) of 4.9. Furthermore, as highlighted previously, the adoption of the NEWS2 score provides an invaluable tool. Its utility is not restricted solely to discerning scoring variances during initial and concluding ED visits. It can also reliably assess septic patients at any stage due to its extensive clinical validation. As discussed above, the adoption of NEWS2 score, not only limited to the scoring difference in the initial and final ED stay, could also be utilized to evaluate the sepsis patients continuously at any phase with confidence because of its wide clinical validation.

In the context of our rese, it was elucidated that interventions during the ED stay had a salient impact on hospital mortality, with an adjusted OR of 0.891. This suggests that approximately a 10% reduction in mortality rate can be attributed to treatments rendered in the ED. Since the introduction of early goal-directed therapy in 1997, efficacious ED treatments have been recognized as instrumental in reducing mortality rates among sepsis patients. Our study corroborates this notion, employing the NEWS2 score as a metric, thereby underscoring the significance of the ED as the initial medical touchpoint for sepsis cases.

### Strengths and limitations

This study, by utilizing the NEWS2 score, demonstrated the contribution of ED management, that may include early diagnosis, fluid resuscitation, antibiotics use, vasopressor or inotropic use, and infection source control, in the treatment course of sepsis. It much explored the use of NEWS2 sore in clinical utilization, especially in evaluating the effect of certain treatment measures. NEWS2 sore, not like the common risk scoring system, such as MEWS or MEDS score, in which only the initial information was collected to predict the final hospital outcome.

There were limitations in this study which should be discussed. First, limited to a medical center study, the study result could not explore to other hospitals in Taiwan or the world [21, 22]. Further validation was needed. Second, the comparison of sepsis definition and treatments, according to the guidelines at that time, was not particularly discussed here since we focused on the utilization of the NEWS2 application for clinical evaluation in improvement or deterioration.

## Conclusion

The present investigation has demonstrated that efficacious treatment of ED, ascertained by means of the NEWS2 scoring system, has led to a reduction in the ultimate mortality rate of patients in the hospital, irrespective of the duration of their stay in the ED. The statement also clarified the importance of ED management in the comprehensive course of sepsis treatment. The practical dynamics of the NEWS2 score have been utilized to clearly depict such intricacies.

### Electronic Supplementary Material

Below is the link to the electronic supplementary material.

Supplementary Material 1

Supplementary Material 2

## Data Availability

The data that support the findings of this study are available from the involved hospitals, mentioned in the manuscript, but restrictions apply to the availability of these data, which were used under license for the current study, and so are not publicly available.

## Abbreviations

AMI: Acute myocardial infarction
CCI: Charlson comorbidity index
CHF: Congestive heart failure
COPD: Chronic obstructive pulmonary disease
CRP: C-reactive protein
ED: Emergency department
ICU: Intensive Care Unit
NEWS2: National Early Warning Score 2
SD: standard deviation
SOFA score: Sequential organ failure assessment score
