# Supplementary material for: Utilizing the National Early Warning Score 2 (NEWS2) to confirm the impact of emergency department management in sepsis patients: a cohort study from taiwan 1998–2020
Source: Int J Emerg Med. 2024 Mar 15;17:42. doi: 10.1186/s12245-024-00614-4 (PMC10941441; doi:10.1186/s12245-024-00614-4)
Supplement: Supplementary file 1 — Supplementary Material 1 [file 12245_2024_614_MOESM1_ESM.docx]

**Supplementary table 1**

| Mortality rate (%) | **Higher CCI score** | **Lower CCI score** |
| --- | --- | --- |
| **Improvement of NEWS 2**  (n = 5,598, 50.84%) | 46.38 % | 30.45 % |
| **Non-improvement of NEWS 2**  (n = 5,403, 49.16%) | 56.50 % | 38.72 % |

Patients were classified into "higher" or "lower" CCI score categories based on the mean CCI score. In the group with NEWS2 improvement, the hospital mortality rate for patients with a higher CCI score was 46.38%, whereas those with a lower CCI score had a mortality rate of 30.45% (P < 0.05). Similarly, in the non-improvement group, patients with a higher CCI score exhibited a mortality rate of 56.50%, in contrast to the 38.72% rate observed in those with a lower CCI score (P < 0.05). (Supplementary table 1)
