# Supplementary material for: Utilizing the National Early Warning Score 2 (NEWS2) to confirm the impact of emergency department management in sepsis patients: a cohort study from taiwan 1998–2020
Source: Int J Emerg Med. 2024 Mar 15;17:42. doi: 10.1186/s12245-024-00614-4 (PMC10941441; doi:10.1186/s12245-024-00614-4)
Supplement: Supplementary file 2 — Supplementary Material 2 [file 12245_2024_614_MOESM2_ESM.docx]

| **Supplementary table 2** | | |
| --- | --- | --- |
| Mortality rate (%) | **Septic shock** | **Non-septic shock** |
| **Improvement of NEWS 2**  (n = 5,598, 50.84%) | 55.52 % | 20.72 % |
| **Non-improvement of NEWS 2**  (n = 5,403, 49.16%) | 64.58 % | 27.36 % |

In the group that showed NEWS2 improvement, septic shock patients had a hospital mortality rate of 55.52%, significantly higher than the 20.72% rate observed in non-septic shock patients (P < 0.05). Similarly, in the non-improvement group, the mortality rate was 64.58% for septic shock patients, in contrast to 38.72% for those without septic shock (P < 0.05). (Supplementary table 2)
